# Classification and Visualization of Alzheimer’s Disease using Volumetric Convolutional Neural Network and Transfer Learning

**DOI:** 10.1038/s41598-019-54548-6

**Published:** 2019-12-03

**Authors:** Kanghan Oh, Young-Chul Chung, Ko Woon Kim, Woo-Sung Kim, Il-Seok Oh

**Affiliations:** 1 Jeonbuk National University, Department of Computer Science and Engineering, Jeonju, 54896 Korea; 2 Research Institute of Clinical Medicine of Jeonbuk National University-Biomedical Research Institute of Jeonbuk National University Hospital, Jeonju, 54907 Korea; 3 Jeonbuk National University Medical School, Department of Psychiatry, Jeonju, 54907 Korea; 4 Jeonbuk National University Medical School, Department of Neurology, Jeonju, 54907 Korea

**Keywords:** Machine learning, Schizophrenia

## Abstract

Recently, deep-learning-based approaches have been proposed for the classification of neuroimaging data related to Alzheimer’s disease (AD), and significant progress has been made. However, end-to-end learning that is capable of maximizing the impact of deep learning has yet to receive much attention due to the endemic challenge of neuroimaging caused by the scarcity of data. Thus, this study presents an approach meant to encourage the end-to-end learning of a volumetric convolutional neural network (CNN) model for four binary classification tasks (AD vs. normal control (NC), progressive mild cognitive impairment (pMCI) vs. NC, stable mild cognitive impairment (sMCI) vs. NC and pMCI vs. sMCI) based on magnetic resonance imaging (MRI) and visualizes its outcomes in terms of the decision of the CNNs without any human intervention. In the proposed approach, we use convolutional autoencoder (CAE)-based unsupervised learning for the AD vs. NC classification task, and supervised transfer learning is applied to solve the pMCI vs. sMCI classification task. To detect the most important biomarkers related to AD and pMCI, a gradient-based visualization method that approximates the spatial influence of the CNN model’s decision was applied. To validate the contributions of this study, we conducted experiments on the ADNI database, and the results demonstrated that the proposed approach achieved the accuracies of 86.60% and 73.95% for the AD and pMCI classification tasks respectively, outperforming other network models. In the visualization results, the temporal and parietal lobes were identified as key regions for classification.

## Introduction

To date, the analysis of neuroimaging data, such as those obtained from magnetic resonance imaging (MRI), positron emission tomography, functional MRI (fMRI), and diffusion tensor imaging, has primarily been performed by experts such as radiologists and physicians, thus requiring a high degree of specialization. Alzheimer’s disease (AD), characterized by the progressive impairment of cognitive and memory functions, is the most common type of dementia that often appears in persons over 65 years old^1^. To slow the progression of dementia, timely treatment is crucial, which requires the early diagnosis of AD and its prodromal stage, mild cognitive impairment (MCI). To this end, a reliable diagnosis from brain imaging is necessary, and a robust diagnostic system aided by the analysis of neuroimaging data would allow for a more informative and reliable approach and could also potentially increase diagnostic accuracy. Traditional analytic methods for exploring neuroimaging biomarkers for the analysis of neuropsychiatric disorders have been based on mass univariate statistics under the assumption that different regions of the brain act independently. However, this assumption is not appropriate given our current understanding of brain functioning^2^.

Recently, machine learning (ML) methods that can take the intercorrelation between regions into account have become an attractive and fundamental element of computer-assisted analytical techniques^3,4^, and has been widely employed for the automated diagnosis and analysis of neuropsychiatric disorders. Although various machine-learning models have been used for automated neurological disorder prediction, two major research directions involve support vector machine (SVM)-based and deep learning (DL)-based diagnosis models. In this regard, extensive reviews related to medical imaging using machine learning techniques have been published^5,6^. SVM-based, automated diagnostic models for neuropsychiatric disorders^7–9^ tend to use hand-crafted features due to their inability to extract adaptive features. The functional connectivity y (FC) patterns representing brain region correlations are a popular feature of existing SVM-based diagnosis models. Individual FC patterns are extracted for pairs of segmented brain patches defined by automated anatomical labeling. Despite its popularity, SVM has been criticized for its poor performance on raw data and for requiring the expert use of design techniques to extract informative features^10,11^.

**Figure 1:**
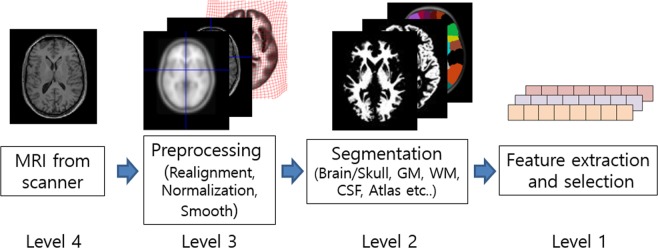
Notion of the end-to-end learning levels.

In contrast, DL models allow a system to use raw data as input, thereby allowing them to automatically discover highly discriminative features in the given training data set^12^. This end-to-end learning design philosophy is the fundamental basis of DL. The primary advantage of end-to-end learning is that all steps in the processing pipeline are simultaneously optimized, potentially leading to optimal performance. This paper proposes an end-to-end hierarchy for the domain of brain MRI analysis, as shown in Fig. 1. The levels of the hierarchy range from 1 (none) to 4 (full). Most of the existing studies use Level 1 or Level 2, whose performances depend heavily on specific software and sometimes even on the hyper parameter setting and manual noise removal. Due to these dependencies, performance evaluation in those studies used only a subset of the original datasets, excluding seeming outliers and making a fair performance comparison difficult. Another advantage of end-to-end learning is that an effective visual explanation for why the CNN reached its classification decision is possible. The explanation helps the doctor to understand the behaviors of the CNNs and to discover new biomarkers. In Level 2, the explanation is limited to the segmented part, which may block the possibility of discovering new biomarkers located in the eliminated region. In Level 1, an explanation is impossible or unreliable since a backward mapping to the 3D space is completely broken at the input level.

Early DL-based approaches^4,13–17^ operate at Level 1 and are far from automatic feature extraction. For example, three-dimensional (3D) or 4D volume data are rearranged into 1D vector form for the use of the DL network’s input, such as restricted boltzmann machine (RBM) and deep belief network (DBN). The reliance on hand-crafted features can be attributed to data scarcity and high dimensionality, which are endemic characteristics of medical data. For example, the ADNI dataset only has several hundred images, while each image has over 11 million dimensions (256 × 256 × 170 voxels). It is critical to note that the aforementioned methods distort neighbor relations (spatial locality) in brain-imaging data during the feature extraction stage. Without preserving spatial relations, it is difficult to expect a reliable explanation of how the network reaches a classification decision.

The convolutional neural network (CNN) has been proven to be a very powerful DL model, which is suitable for grid-like data such as RGB images and MR images. Starting with the great success of AlexNet on the natural image classification problem^18^, the application of CNNs has rapidly expanded into diverse fields. In medical image analysis, early successful adoptions were achieved in 2D images such as retinal and chest X-ray images^19^ and then extended to 3D images such as MRI. Existing CNN-based methods for MRI typically belong to Level 2. Some studies^20,21^ segment the gray matter (GM) region during preprocessing and then employ it as an input of the CNN. Rieke *et al*.^22^, Yang *et al*.^23^, and Korolev *et al*.^24^ presented 3D-CNN–based approaches with several regularization techniques, such as dropout (DO), batch normalization (BN), and residual module. Although these approaches yielded impressive results by adopting appropriate regularization techniques, no unsupervised learning was involved. In the field of DL, unsupervised learning is regarded as an essential element when faced with data scarcity and high dimensionality. Strategies based on scratch training may cause bias by initializing the weights randomly for limited amounts of data. Hosseini-Asl *et al*.^25^, whose method belongs to Level 3, reported the first successful application of a volumetric CNN-based architecture on MRI data by employing 3D-stacked Convolutional Autoencoders (3D-sCAE) for the classification of AD vs. NC; in their method, a pretraining model to learn sparse representations and fine-tuning techniques are used to construct the final supervised network models. However, our reproduction of their model achieved only 80% accuracy on AD vs. NC classification. Furthermore, there was no attempt to explain how the classification decision was reached.

Multimodal DL approaches^4,16,17,26–28^ have attempted to incorporate different inputs and DL models to improve the classification accuracy of AD. Lee *et al*.^16^ extracted multimodal features, reflecting MRI, Cohort data and CSF data, and employed an RNN to predict AD. Additionally, Suk *et al*.^27^ employed MRI, PET, MMSE and CSF data to classify AD vs. MCI. Feng *et al*.^28^ proposed an incorporated framework for 3D-CNN and LSTM and fed MRI and PET data together to the network. They showed that multimodal data are effective in improving classification performance.

Some of the studies presented visual explanations. In particular, Yang *et al*.^23^ and Rieke *et al*.^22^ focused on an explanation of 3D-CNNs by applying several visual explanation methods, illustrating patchwise prediction differences and Grad-CAM^29^. Although they were able to illustrate how the CNNs arrived at the classification decision, no attempt has yet been made to explain classification task of progressive MCI (pMCI) vs. stable MCI (sMCI).

From a clinical viewpoint, the diagnosis of pMCI patients, who are at risk for progression to dementia, is crucial for the effective treatment of early AD. Despite its importance, few studies^20,30,31^ have addressed the problems of MCI classification. Cheng *et al*.^31^, whose method operates at Level 1, presented the advantage of transfer learning for the classification of pMCI vs. sMCI. In their study, an AD vs. NC task was considered as source domain data. A recent study^20^ also proposed a CNN-based transfer learning scheme using the GM region and showed that transfer learning is useful for CNN-based studies at Level 2. However, their effort to find important biomarkers only showed the mean activation map at the outputs of the first convolutional layer rather than using a backpropagation-based explanation model. Since the stream of the important weights derived from the target output node was ignored, this explanation is limited.

The aims of this study are to develop a volumetric CNN-based approach for AD classification and to visualize the spatial attention of the CNN’s decision to discover important biomarkers related to AD in an end-to-end learning manner (Level 3). The main contributions and characteristics of this study can be summarized in detail as follows: (1) We proposed a notion of the end-to-end hierarchy for MRI classification problems and proposed a method operating at level 3. For successful learning without overfitting at Level 3, we adopted several schemes for data augmentation and regularization. A transfer learning scheme for the pMCI classification that transfers the weights learned during AD vs. NC to pMCI vs. sMCI is presented. (2) We applied a gradient backpropagation-based visualization technique to learned models and identified important biomarkers from these much cleaner visualization results. To the best of our knowledge, this is the first successful explanation of CNNs for the classification of pMCI with end-to-end learning.

## Methods

**Figure 2:**
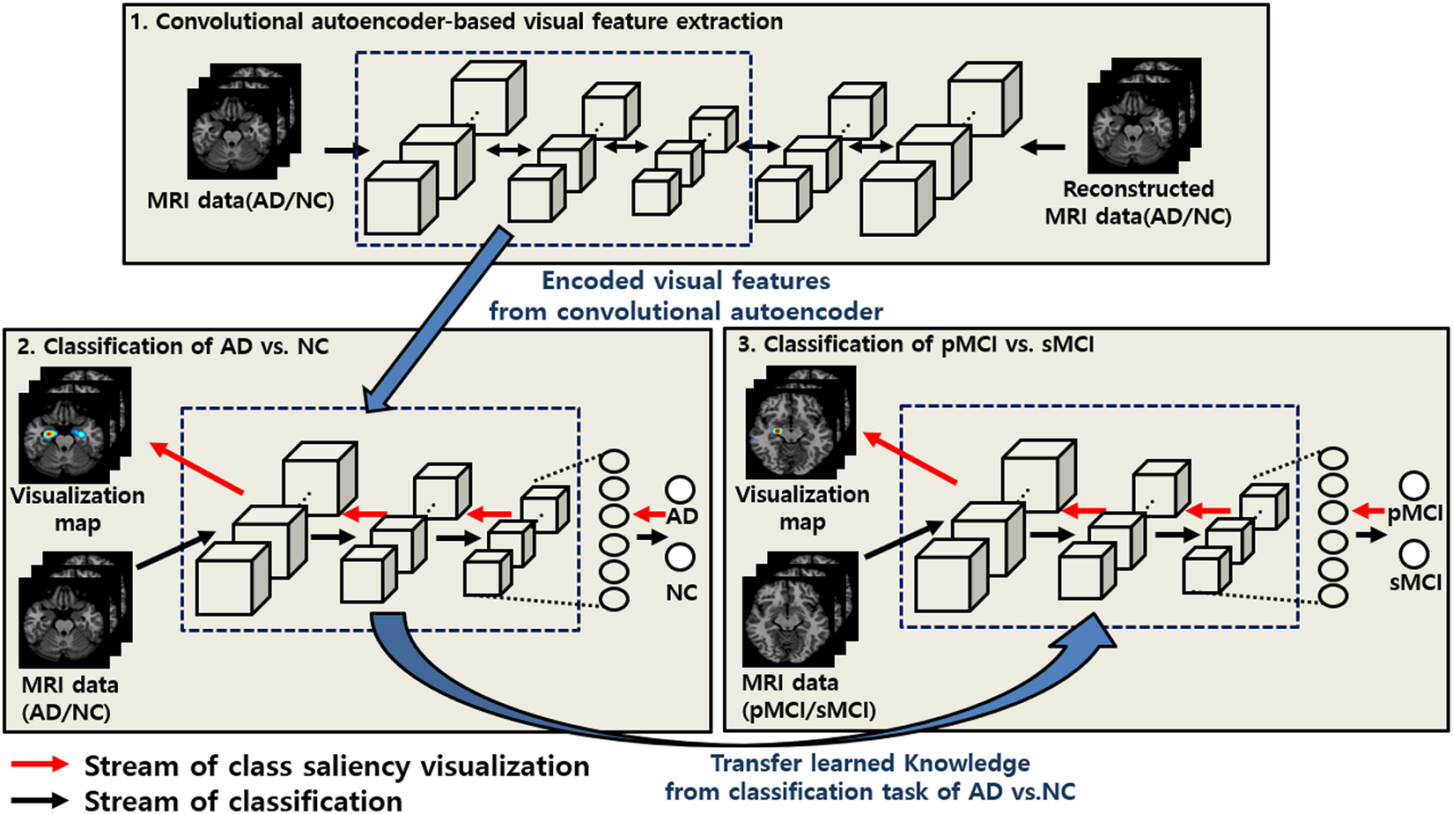
Overall architecture of the proposed approach. (1) CAE-based unsupervised learning is conducted to extract sparse representations; (2) the AD vs. NC classifier is constructed through supervised fine-tuning; (3) to construct the pMCI vs. sMCI classifier of pMCI vs. sMCI, the representations of the AD vs. NC task are considered as initial weights under a transfer learning manner. Finally, the important biomarkers are localized using a gradient-based approach.

The schematic diagram of the proposed approach for classifying neuroimaging data is summarized in Fig. 2 and presented in detail in this section. First, MRI scans of patients with AD and of NCs were pretrained based on CAE-based unsupervised learning, and then, supervised fine-tuning was conducted to build the classifier to differentiate AD vs. NC. Second, since the pMCI vs. sMCI classification task is more difficult and subtler than the classification of AD/NC, we employed a transfer learning procedure, for which the visual representations drawn from the classification task of AD/NC were transferred to the pMCI vs. sMCI learning model to improve the generalization capability of the pMCI vs. sMCI classification task. Finally, the most important biomarkers on each classification task were detected using the class saliency visualization technique.

### ADNI data and preprocessing

In this study, we use the Alzheimer’s Disease Neuroimaging Initiative (ADNI) dataset, which is publicly available on the web (http://adni.loni.usc.edu/). The ADNI aims to identify more sensitive and accurate methodologies for diagnosing Alzheimer’s disease at earlier stages as well as mark the progress of AD through biomarkers. In this work, we used a total of 694 structural MRI scans that were initially classified into AD (n = 198), NC (n = 230), pMCI (n = 166), and sMCI (n = 101) at baseline. The 166 pMCI subjects were initially diagnosed with MCI at baseline, but conversion to AD was reported within a 36-month follow-up. The subjects were between 55 and 90 years old, and the ranges of MMSE scores for each group were 20–26 (AD), 24–30 (MCI), and 24–30 (NC). The clinical dementia rating (CDR) scores were 0 for the NC subjects, 0.5 and with a mandatory memory box score of at least 0.5 for the MCI subjects, and 0.5 or 1 for the AD subjects. Given the single modality of sMRI, most of the available sMRI scans in the ADNI database were used, except for the scans of unknown MCI (n = 130), in which the subjects were missing a diagnosis at 36 months from the baseline. The sMRI scans (T1-weighted MP-RAGE sequence at 1.5 Tesla) typically consisted of 256 × 256 × 176 voxels with a size of approximately 1 mm × 1 mm × 1.2 mm. The characteristics of the subjects included in this study and the p-values of the t-test comparisons for each group are summarized in Table 1. For preprocessing, all T1-weighted scans were corrected for bias-field inhomogeneities prior to being downloaded as a part of the ADNI preprocessing protocol. The MRI scans were then spatially normalized to the MNI space using Statistical Parametric Mapping (SPM12; HTTP://WWW.FIL.ION.UCL.AC.UK/SPM/) and the Diffeomorphic Anatomical Registration Exponentiated Lie Algebra (DARTEL) registration method^32^. The dimensions after spatial normalization were uniformly rescaled to 120 × 160 × 120 for CNN learning. The intensities of the MRI scans were normalized to [0 1]. As previously mentioned, unlike most of the existing approaches, no skull striping and segmentation methods, which are heuristic and fragile, were used to maximize the capability of end-to-end learning. Note that in this study, the spatial normalization must be performed to address the proposed visualization process for which a statistical saliency map is computed.

**Table 1:** Clinical characteristics of the subjects.

Category	AD	NC	pMCI	sMCI	*P*, AD vs NC	*P*, pMCI vs sMCI	*P*, NC vs pMCI	*P*, NC vs sMCI	*P*, AD vs pMCI	*P*, AD vs sMCI
Number	198	230	166	101						
Female/male	97/101	112/118	103/63	35/66	0.968^*^	0.631^*^	0.028^*^	0.018^*^	0.044^*^	0.027^*^
Age (mean ± SD)	75.65 ± 7.76	75.98 ± 5.00	74.85 ± 7.71	74.15 ± 7.66	0.585	0.721	0.230	0.206	0.604	0.445
MMSE (mean ± SD)	23.39 ± 2.04	29.12 ± 0.99	26.45 ± 2.81	28.19 ± 1.71	<0.001	<0.001	<0.001	<0.001	<0.001	<0.001
GlobalCDR (mean ± SD)	0.76 ± 0.25	0.00 ± 0.00	0.50 ± 0.00	0.50 ± 0.00	<0.001	—	—	—	—	—

*P according to chi-square test.

### Convolutional autoencoder based unsupervised learning

**Figure 3:**
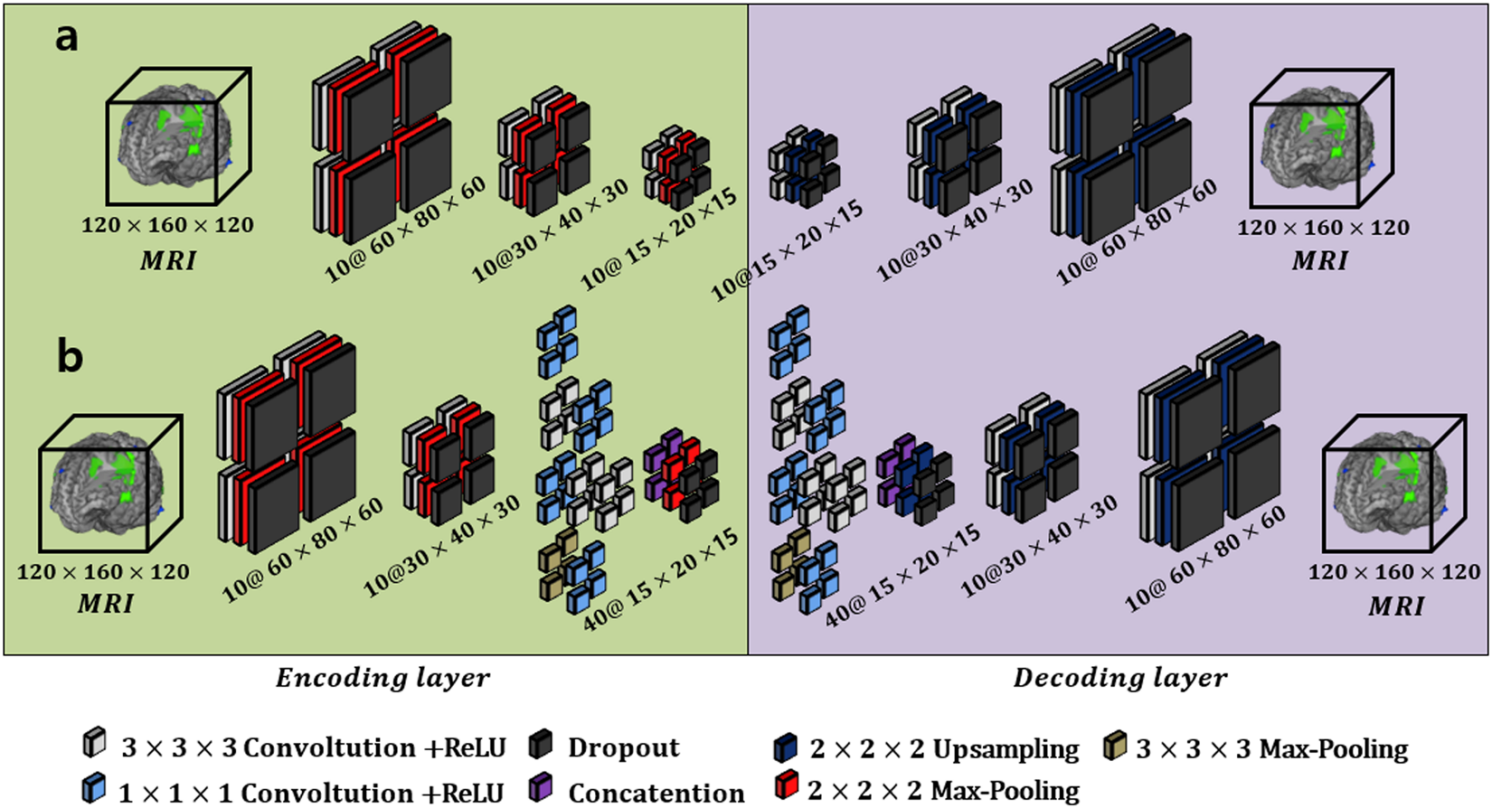
Convolutional autoencoder-based unsupervised learning. Architecture (**a**) represents the traditional CAE, and the inception module is applied to architecture (**b**).

Autoencoders are unsupervised neural network models whose main purposes typically include dimensionality reduction and information retrieval tasks. In particular, dimensionality reduction was one of the first applications of representation learning and DL, and discriminative representations in a lower-dimensional space can improve the performance of a classification task^33,34^. Moreover, this process does not require labeled data. However, AEs, which ignore the spatial structure of an image, are unsuitable for analyzing 2D- or 3D-image data because the effectiveness of localized spatial features has already been demonstrated by successful object-image-analysis models^35^. Convolutional Autoencoders (CAE), which extend AEs, have been employed for image data analysis and generally comprise the following encoding and decoding steps: input data are transformed into a lower-dimensional feature space during the encoding phase, and then the encoded data are reconstructed in the original space in the output layer during the decoding phase. For a 3D tensor ${\boldsymbol{X}}$ (of size h × w × d), the encoder is defined as follows:

1 $${{\boldsymbol{ {\mathcal H} }}}_{i,k}={\mathscr{D}}({\rm{\varphi }}(\sigma ({{\boldsymbol{W}}}_{i,k}\ast {\boldsymbol{X}})))$$

where ${{\boldsymbol{W}}}_{i,k}$ is the *i*-type 3D filter (*i.e*., the case in which an inception module has multiple types of convolutional filters) for the *k*-th feature tensor, * denotes 3D convolution, and ${\mathscr{D}}(\,\cdot \,)$ and *σ*(⋅) denote the dropout and the elementwise nonlinear activation functions (ReLU [10] in this paper), respectively. Following the encoding phase, the decoder can be defined as follows:

2 $${\boldsymbol{X}}{\boldsymbol{^{\prime} }}={\mathscr{D}}({\rm{\varphi }}^{\prime} (\sigma (\sum {\sum }_{i{\epsilon }{\mathbb{T}},k{\epsilon }{\mathbb{F}}}{{\boldsymbol{W}}}_{{\boldsymbol{k}}}^{\text{'}}\ast {{\boldsymbol{ {\mathcal H} }}}_{i,k})))$$

where ${\boldsymbol{X}}{\boldsymbol{^{\prime} }}$ denotes the 3D reconstructed map and ${{\boldsymbol{W}}}_{{\boldsymbol{k}}}^{\text{'}}$ is the 3D filter for the decoding process. From Eq. (1), ${\mathbb{T}}$ is the group of filter types, ${\mathbb{F}}$ represents the group of feature tensors corresponding to each filter type, and φ and φ′ are max-pooling and upsampling, respectively. The loss function used to minimize the error between the input, ${\boldsymbol{X}}$, and the reconstructed input, ${\boldsymbol{X}}{\boldsymbol{^{\prime} }}$, is the cross entropy:

3 $$E(\theta )=-\mathop{\sum }\limits_{k=1}^{N}\,[{{\boldsymbol{X}}}_{i}log\,{{\boldsymbol{X}}}_{i}^{^{\prime} }+(1-{{\boldsymbol{X}}}_{i})log(1-{{\boldsymbol{X}}}_{i}^{^{\prime} })]$$

where *N* is the number of training samples and $\theta =\{{\boldsymbol{W}},{\boldsymbol{W}}{\boldsymbol{^{\prime} }}\}$ denotes the set of trainable parameters. Finally, the error given by Eq. (3) is minimized using the Adam optimizer^36^, and the weight parameters for the layers are updated by backpropagation. Unlike the case of 2D convolution, the 3D feature space is explored by moving the 3D convolutional filters, resulting in 4D feature tensors (in the 2D case, convolutional filters are used to construct the 3D tensors). In this work, we constructed two types of models, traditional and inception module-based CAE models, and their architectures are illustrated in Fig. 3(a,b), respectively. Each of the proposed *Conv* layers comprises the following components: convolution, dropout, ReLU, and pooling (or upsampling). Note that we employed a padding function to preserve the original scale. In the ICAE approach, the third and fourth *Conv* layers were replaced with the inception module.

### Inception module for the convolutional autoencoder

**Figure 4:**
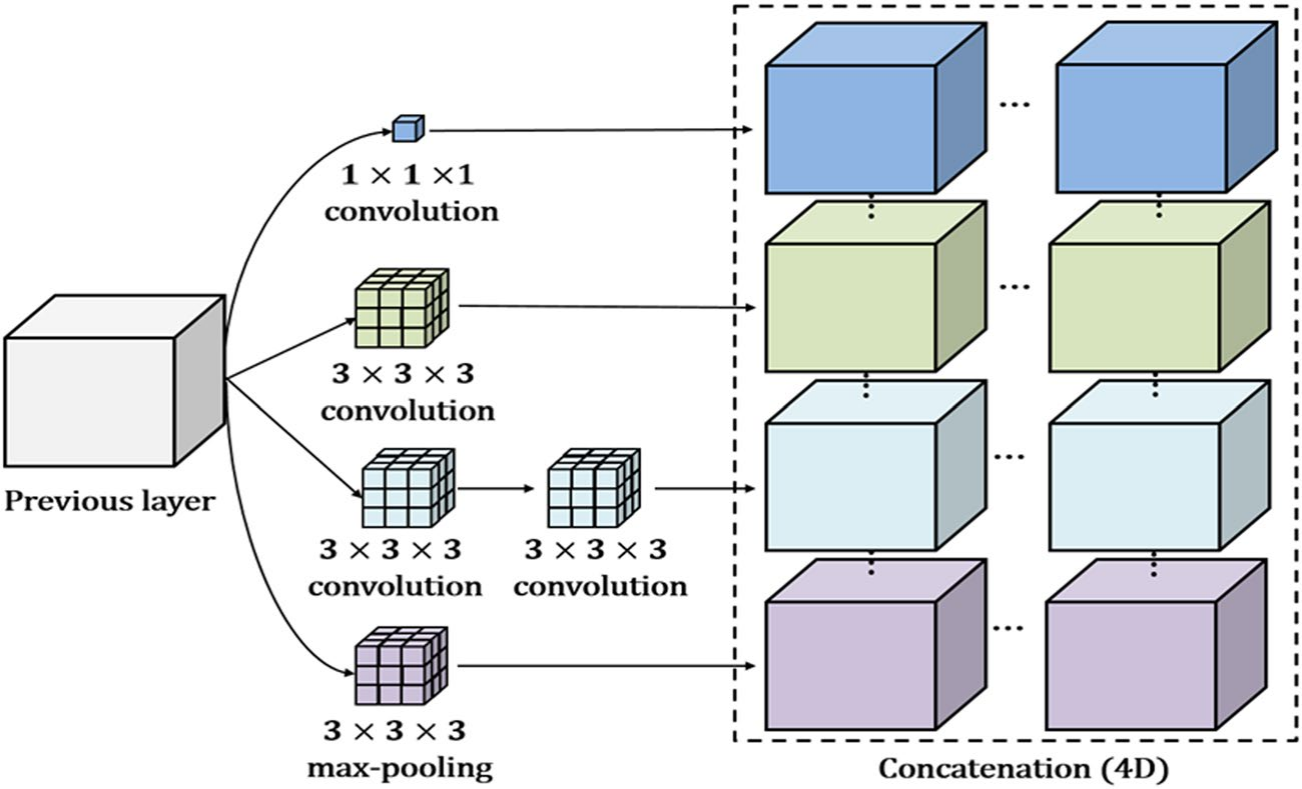
Example of the inception module for a 3D tensor.

In an attempt to discover highly advanced representations and reduce dependency on the fully connected (FC)-layer, we applied an inception module to the convolutional autoencoder. Note that the FC layer typically contains a large number of parameters, resulting in overfitting. Our inception module is based on GoogLeNet inception version 2^37^, which is a parallel structure comprising differing 3D convolution, pooling filters, and concatenation. Rather than using a single convolution, the inception module extracts multilevel representations over the same input tensor in parallel and concatenates the results into a single output tensor. As shown in Fig. 4, since our model is based on a 3D tensor, the result of the inception module is a 4D, concatenated, single tensor. The entire architecture of the inception module-based CAE (ICAE) is illustrated in Fig. 3(b). The ICAE mainly comprises three encoding and decoding phases, with each proposed network module receiving its input based on the output of the previous module. We included two stem layers with traditional structures (convolution + pooling) in both the encoder and decoder layers, similar to the arrangement found in GoogLeNet^37^. For calculation convenience, we employed a padding function to preserve the original scale in the convolution and pooling processes of each inception module. Therefore, a scale reduction was accomplished by pooling operations following the inception modules (or convolution). To increase computational efficiency, we replaced the 5 × 5 × 5 convolution block with two layers of 3 × 3 × 3 convolution blocks, and a 1 × 1 × 1 filter was employed to reduce a dimension of the feature map prior to the expensive 3 × 3 × 3 and 5 × 5 × 5 convolutions. In addition, the strategy by which 1 × 1 convolutions are followed by classic convolutions is known to increase the nonlinearity of the learning model compared to conventional convolutional layers, thus helping detect more highly advanced visual representations than the previous strategy.

### Fine-tuning the convolutional neural network

Unsupervised learning procedures are typically used to find a good initialization for joint training over all layers for the supervised task^38,39^. In the proposed method, we employed a task specific fine-tuning technique^40,41^ using predefined initial weights drawn from CAE-based unsupervised learning rather than randomly initializing the trainable weights. To this end, the first three *Conv* layers of the 3D-CNNs were initialized using the pretrained weights from the encoding layers of the abovementioned CAE (Fig. 3), and the supervised fine-tuning of CNNs was performed for each task-specific binary classification. In general, the network architecture implements the following two main phases: a) generic features from neuroimaging data are extracted in the convolutional layer and b) the extracted generic features are classified into AD vs. NC and pMCI vs. sMCI in the classification layer. This strategy is able to partially overcome the overfitting problem caused by the limited amount of data as well as by data with high dimensionality.

### Transfer learning for MCI classification

Research involving transfer learning is motivated by the fact that people can intelligently utilize related knowledge that has been previously learned in solving new problems faster or finding better solutions^41,42^. In transfer learning, the learning must be performed in two or more tasks, and this multitask learning can be achieved by representation learning when useful features can be extracted from the related domain. This is typically regarded as a supervised learning context where the input is the same, but the target task may be of a different nature^34^. Recently, transfer learning has become very popular in the field of DL, since it has been shown to be a powerful methodology that enables DL training with comparatively insufficient data. For example, if there is sufficiently more data in the first task in source domain ${{\mathbb{R}}}_{1}$, it may help to quickly improve the generalization capability of the learning model in the second task, even if it includes little data, in target domain ${{\mathbb{R}}}_{2}$. This is because many visual representations share low-level generic notions, such as edges, visual shapes, changes in intensity or lighting, and the effects of geometric changes. In this study, the amount of MRI data for the second task (pMCI vs. sMCI) is relatively smaller than that for the first task (AD vs. NC); additionally the second task has been known to be a more difficult problem than the first task. Fortunately, it is known that both tasks typically share significant biomarkers^43,44^ and references therein. Based on this observation, we thus employed a knowledge transferring technique to solve the pMCI vs. sMCI classification problem rather than considering an unsupervised learning approach. In this part of the section, we indicate some notations and definitions of transfer learning in reference to a survey study^45^. A domain ${\mathbb{D}}$ is comprised of two factors, a feature space ${\mathbb{X}}$ and a probability distribution *P* (*X*), where $X=\{{{\boldsymbol{X}}}_{1},{{\boldsymbol{X}}}_{1},\,\ldots ,\,{{\boldsymbol{X}}}_{n}\}\in \,{\mathbb{X}}$. In this study, ${\mathbb{X}}$ can be considered as the feature space of all possible MRI data in the specific domain ${\mathbb{D}}$; then, *x*_*i*_ is the *i*^*th*^ MRI data (instance), and *X* is a particular class. Given some domain ${\mathbb{D}}$ = {${\mathbb{X}}$, *P*(*X*)}, a task ${\mathbb{T}}$ consists of two factors, a label space ${\mathbb{Y}}$ and a predictive function $f(\,\cdot \,)$ that is trained by the representation of the MRI and the corresponding label pairs {*x*_i_, *y*_i_}, where *x*_i_ ∈ ${\mathbb{X}}$, *y*_i_ ∈ ${\mathbb{Y}}$. The function $f(\,\cdot \,)$ is employed to classify new instances into their corresponding labels and can be written as *P*(*y*|*x*). Note that the volumetric CNN can be considered the predictive function $f(\,\cdot \,)$ in our implementation. Now, we define a domain ${\mathbb{D}}$ = {${\mathbb{X}}$, *P*(*X*)} and a task ${\mathbb{T}}=\{{\mathbb{Y}},f(\,\cdot \,)\}$. For transfer learning, we used the AD vs. NC problem as the source domain data, where ${{\mathbb{D}}}_{{\rm{AD}}}$ = {(*x*_*AD*1_, *y*_*AD*1_), …, (*x*_*ADn*_, *y*_*ADn*_)}. In the same manner, the target domain data reflecting the pMCI vs. sMCI problem can be written as ${{\mathbb{D}}}_{{\rm{pMCI}}}=\{({x}_{pMCI1},{y}_{pMCI1}),\,\ldots ,\,({x}_{pMCIn},{y}_{pMCIn})\}$. In the following description, the source and target tasks are defined as ${{\mathbb{T}}}_{{\rm{AD}}}=\{{{\mathbb{Y}}}_{{\rm{AD}}},{f}_{{\rm{AD}}}(\,\cdot \,)\}$ and ${{\mathbb{T}}}_{{\rm{pMCI}}}=\{{{\mathbb{Y}}}_{{\rm{pMCI}}},{f}_{{\rm{pMCI}}}(\,\cdot \,)\}$, respectively. We now provide a definition of transfer learning for the detection of pMCI.

#### Definition 1.

Given a source domain ${{\mathbb{D}}}_{{\rm{AD}}}$ with its task ${{\mathbb{T}}}_{{\rm{AD}}}$ and a target domain ${{\mathbb{D}}}_{{\rm{pMCI}}}$ with its task ${{\mathbb{T}}}_{{\rm{pMCI}}}$, transfer learning aims to boost the generalization capability of the target predictive function ${f}_{pMCI}(\,\cdot \,)$ through the transfer of knowledge from ${{\mathbb{D}}}_{{\rm{AD}}}$ and ${{\mathbb{T}}}_{{\rm{AD}}}$, where ${{\mathbb{D}}}_{{\rm{AD}}}\ne {{\mathbb{D}}}_{{\rm{pMCI}}}\,{\rm{or}}\,{{\mathbb{T}}}_{{\rm{AD}}}\ne {{\mathbb{T}}}_{{\rm{pMCI}}}$.

In implementing the MCI classification model, a set of trainable weights for the first three *Conv* layers are denoted as ${\theta }_{pMCI}=\{{{\boldsymbol{W}}}_{pMCI},{{\boldsymbol{b}}}_{pMCI}\}$, and the set of weights *θ*_*pMCI*_ is replaced with those of the pretrained weight set ${\theta }_{AD}=\{{{\boldsymbol{W}}}_{AD},{{\boldsymbol{b}}}_{AD}\}$ that is trained from the AD vs. NC problem, where ${\theta }_{pMCI}\leftarrow {\theta }_{AD}$; then, joint supervised learning is implemented over all of the layers to construct the pMCI vs. sMCI classifier.

### Class saliency visualization approach

In this section, we describe a class saliency visualization (CSV) method^46^ for approximating the spatial influence of a DL model’s decision on a given target class. The CSV method calculates how much each input voxel ${{\boldsymbol{X}}}_{ijk}$ contributes to the final activation of the target class. Given an MRI data ${\boldsymbol{X}}$, a target class *c*, and a classification model, the method computes the voxelwise saliency scores of ${\boldsymbol{X}}$ based on the influence on the prediction score ${f}_{c}({\boldsymbol{X}})$. A motivational example of the CSV method is expressed as ${f}_{c}({\boldsymbol{X}})={\omega }_{c}^{T}\,{\boldsymbol{X}}+{b}_{c},$ where the function ${f}_{{\rm{c}}}({\boldsymbol{X}})$ represents a simple linear model, which computes a score of specific class *c*, and ω_c_ and *b*_*c*_ denote the weight vector and the bias of the model, respectively. Because the important regions of ${\boldsymbol{X}}$ are generally linked by larger weights, the magnitude of *ω* can be considered the saliency score of the CNNs’ decision. Since CNNs are more complex nonlinear models than the motivational example, the first-order Taylor expansion is employed to approximate ${f}_{c}({\boldsymbol{X}})\approx {\omega }^{T}{\boldsymbol{X}}+b$. The class saliency map ***M***, which has the same scale as the original input ${\bf{X}}$, is calculated by the partial derivatives:

4 $${{\boldsymbol{M}}}_{ijk}=|{\omega }_{h(i,j,k)}|=|\frac{\partial {f}_{c}}{\partial {{\bf{X}}}_{ijk}}|$$

Since each voxel in the MRI is connected by at least one weight, the saliency map can be expressed as *ω*_*h*(*i*,*j*,*k*)_, where *h*(*i*, *j*, *k*) denotes the index of the ω that spatially corresponds to MRI voxel ${{\boldsymbol{X}}}_{ijk}$. For this reason, the 3D saliency map can be obtained by rearranging vector *ω*, which is calculated by backpropagation. To construct a visually pleasing saliency map, we applied a 9 × 9 × 9 smoothing filter to the visualization results. In the code of Algorithm 1, we present the proposed procedure for generating the saliency map. Given classifier *f*, target class c, and the corresponding MRI scan ${\boldsymbol{X}}$, instancewise saliency maps were calculated using the CSV approach; then, after applying a smoothing filter, the entirety of the saliency maps were linearly integrated and normalized to obtain the final explainable saliency map. Note that this model requires no additional annotation, such as a segmentation mask or bounding box, to localize the important regions of the neuroimaging data because it directly produces saliency voxels by interpreting the pretrained CNN model. This approach is useful in neuroimaging domains in which the differentiating characteristics of the various disease classes may not be well established by helping to identify meaningful findings in the neuroimaging data that can be used to guide practicing physicians.

**Algorithm 1:**
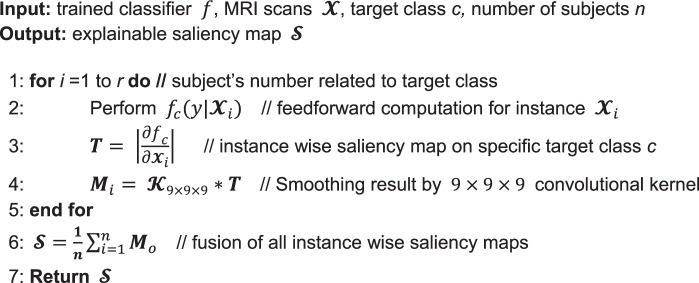
Generation of explainable saliency map.

### Experimental setup

The evaluation of the proposed and competing methods is conducted on four binary autodiagnostic problems: (1) AD vs. NC, (2) pMCI vs. NC, (3) sMCI vs. NC, (4) pMCI vs. AD, (5) sMCI vs. AD, and (4) pMCI vs. sMCI classifications. Due to the limited amount of data, data augmentation was performed. The input MRIs are randomly rotated by [−5, 5], shifted by [0, 0.1] and rescaled by [0.8, 1.2]; second, we perform the intensity changes by randomly adjusting brightness, contrast, saturation and hue. The augmentation operations are based on a uniform distribution.

**Figure 5:**
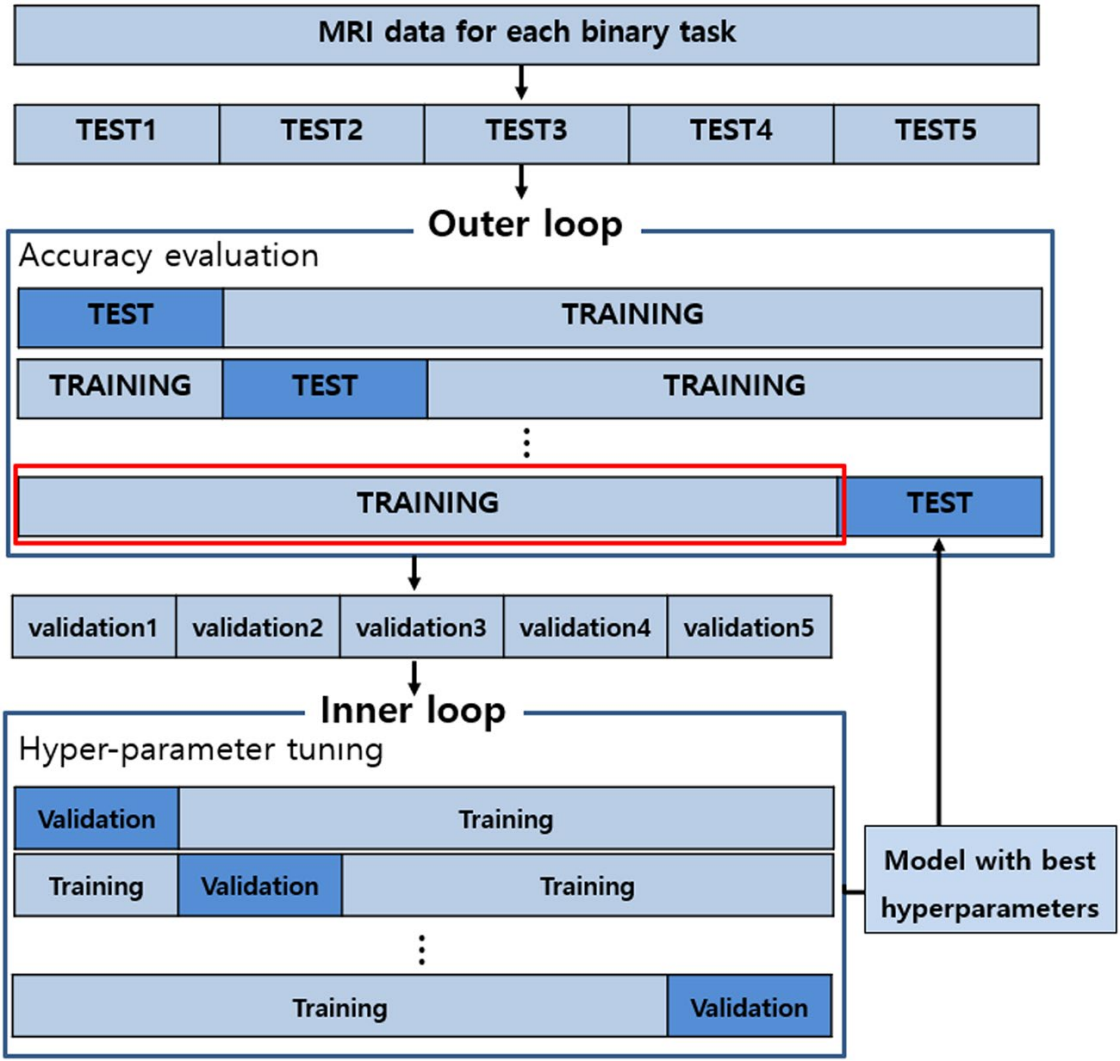
Flowchart of the evaluation procedure using k-fold cross validation.

The evaluation procedure is carried out using nested five-fold cross-validation described in Fig. 5. In this study, two important hyperparameters, DO ratio and the L_1_/L_2_ weighting factors, were optimized using the grid search technique. The nested cross-validation consists of outer and inner procedures. First, an inner cross-validation is used to tune the hyperparameters and select the best model. Second, an outer cross-validation is used to evaluate the model selected by the inner cross-validation. To avoid possible bias caused by the random partitioning of the datasets, the five-fold nested cross-validation is repeated 20 times independently, and the average is reported. Since the same MRI data must be considered for fair performance comparisons, we compare the proposed model with the state-of-the-art model using the same training and testing datasets from the ADNI database. The performance is evaluated via the following quantitative metrics: accuracy (ACC) = (TP + TN)/(TP + TN + FN + FP), sensitivity (SEN) = TP/(TP + FN), specificity (SPE) = TN/(TN + FN), area under the receiver operating characteristic curve (AUC), and balanced accuracy (BAC) = (SEN + SPE)/2. TP, TN, FP, and FN denote the numbers of true-positive, true-negative, false-positive, and false-negative classification results, respectively. Here, if an AD patient is classified into the AD group, this is regarded as a TP and otherwise as an FN. Likewise, TN represents the number of normal subjects classified into the normal group, and FP represents the number of those who are not.

### Details of learning models

For the baseline models, we consider existing models^22,24,25^ that are based on volumetric CNN architectures. Unlike most hand-crafted feature-based diagnostic methods, the baseline models encourage end-to-end learning by preserving spatial information from the neuroimaging data. In terms of methodology, the baseline models are closely related to the proposed approach. The details of the baseline models are as follows: (1) the baseline model presented by Hosseini-Asl *et al*.^25^ is built upon unsupervised learning with a stacked CAE (sCAE) and supervised learning with CNNs, and the architecture is comprised of three 3 × 3 × 3 *Conv* layers with 10 feature maps each, two FC layers with 128 and 64 nodes, and an output layer with two feature maps; (2) in the existing study^24^, a residual neural network^47^-based model was applied. This architecture is comprised of 21 layers containing six residual modules: first, three 3 × 3 × 3 *Conv* layers with 32 feature maps each, then an FC layer with 128 nodes, and finally an output layer with two feature maps; (3) the architecture proposed by Korolev *et al*.^24^ is comprised of four *Conv* layers with 8–16–32–64 feature maps sequentially, batch normalization^40^ and 2 × 2 × 2 max-pooling after each convolution, dropout of 0.8 before the first FC layer, two FC layers with 128 and 64 nodes, and an output layer with two feature maps. For the implementation of the baseline models^22,24,25^, we used the hyperparameters as described therein.

In the proposed approach, we constructed CAE- and ICAE-based network models, and the overall architectures are as follows: (1) The CAE-based model is comprised of three 3 × 3 × 3 *Conv* layers with 10 feature maps each, two FC layers with 32 and 16 nodes, and an output layer with two feature maps; each of the Conv layers is followed by ReLU and 2 × 2 × 2 max-pooling; Gaussian dropout^48^ with a dropping ratio 0.8 is applied between the *Conv* layers; and in the FC and output layers, we used scaled exponential linear unit (SELU) activation^49^ to speed up learning and take a normalization effect, resulting in the CAE-based network having a total of 1.44 million parameters. (2) The ICAE-based model comprises two 3 × 3 × 3 *Conv* layers with 10 feature maps each, a single *inception* module with 40 feature maps, and an output layer with two nodes; in this network, the results of the inception layer are directly fed to the output layer without the FC layer; each of the Conv layers is followed by ReLU and 2 × 2 × 2 max- pooling, and the *inception* module contains multilevel convolutional kernels of different sizes (3 × 3 × 3, 1 × 1 × 1) and stacks their output feature maps in a channel domain to extract visual representations at different scales. We also applied Gaussian dropout between *Conv* and *inception* layers, and SELU activation was considered before the soft-max. The ICAE-based network has a total of 0.34 million parameters. In both the CAE- and ICAE-based models, the unsupervised learning technique (Convolutional autoencoder) is applied before supervised learning to extract a sparse visual representation of neuroimaging data. In the supervised learning, the Adam optimizer was established with a mini-batch size of 10, β_1 = 0.9, and β_2 = 0.9, an initial learning rate of 1 × 10^−4^, and a weight decay of 1 × 10^−5^. In the experiment, most cases reached a convergence state within 200 training epochs, and we considered this as a performance evaluation during the inner cross-validation.

## Results

### Classification performances

**Figure 6:**
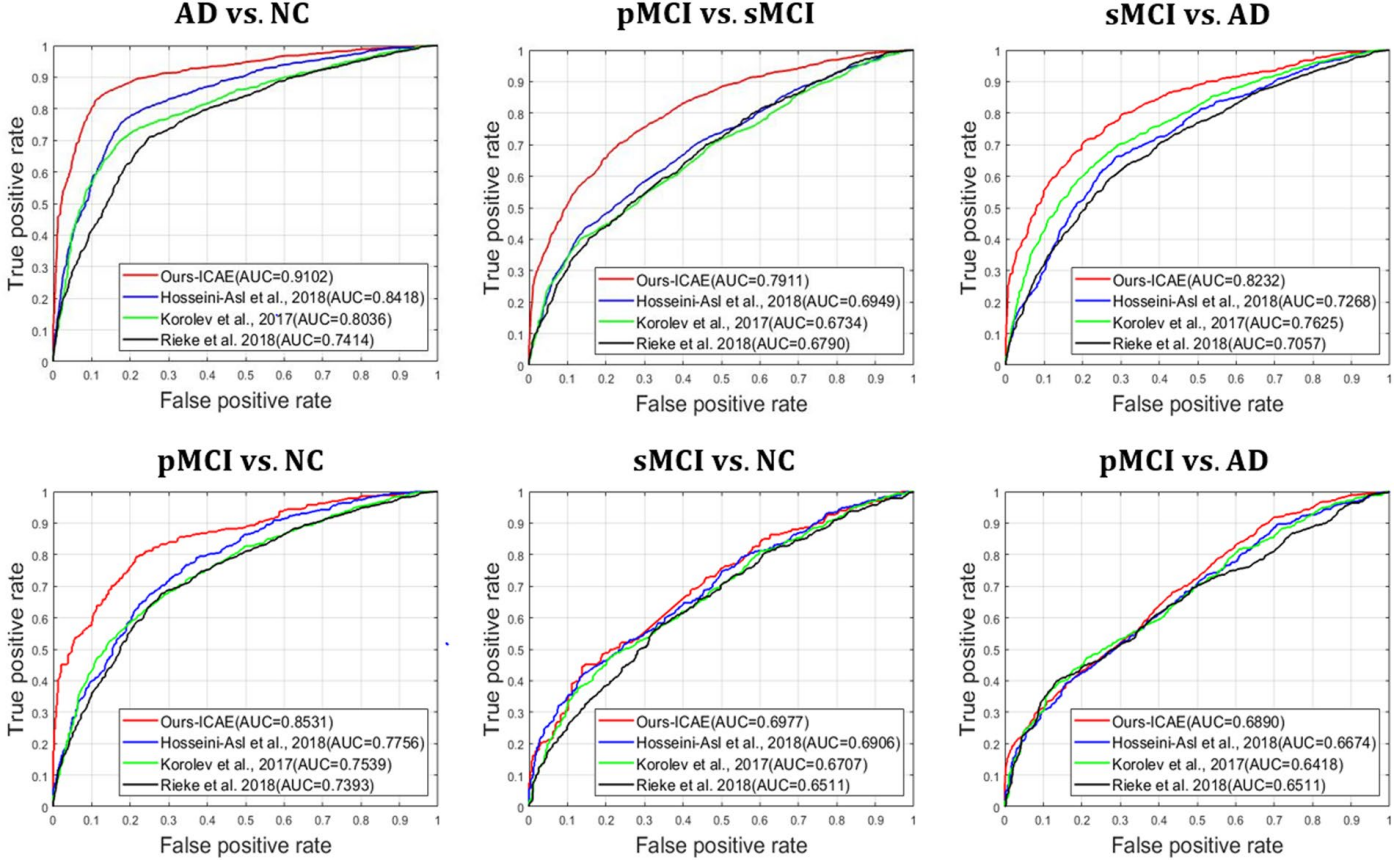
Quantitative performance comparisons of our model and the existing models using ROC curves and AUC. From left to right: (top) AD vs. NC, pMCI vs. sMCI, sMCI vs. AD; (bottom) pMCI vs. NC, sMCI vs. NC and pMCI vs. AD.

**Figure 7:**
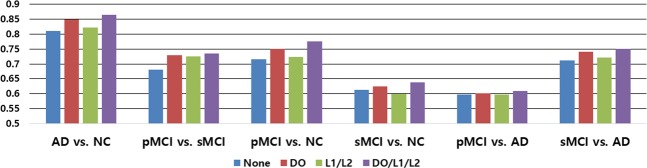
Performance comparisons according to regularization techniques. “None” represents the results without regularization techniques (DO/L1/L2).

**Figure 8:**
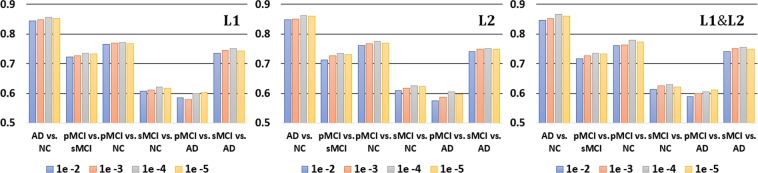
Performance comparisons according to different L1/L2 parameters.

In Table 2, the worst results were observed for CNNs (Scratch), for which the pretraining technique was not considered. This clearly shows the importance of AE-based unsupervised learning, which helps a model learn sparse representations in high-dimensional space, before applying supervised learning. For the unsupervised learning-based models, we set up the following experiments: AD vs. NC, pMCI vs. NC, sMCI vs. NC, pMCI vs. AD and sMCI vs. AD. The results of the CAE- and ICAE-based models showed better performances, which were improved by 5~12% and 7~14% compared to the baseline models^22,24,25^, respectively. Additionally, we measured the receiver operating characteristic (ROC) curves of our model and of the existing models, and our curve is consistently higher than those of the other models (Fig. 6). In Fig. 7, the use of the regularization techniques reflecting L_1_, L_2_ and DO improved the classification accuracy by 3~6% and 4~8% in the CAE and ICAE approaches, respectively. The accuracy changes according to the L_1_/L_2_ regularization methods and their parameters are described in Fig. 8. During the cross-validation, the pentameters except L_1_/L_2_ were fixed to verify their effects. The results show favorable performances when applying both the L_1_ and L_2_ methods together, and the parameters between 1 × 10^−3^~1 × 10^−4^ outperform those of the other cases regardless of various changes in experimental condition.

**Table 2:** Classification performances (%).

Task	Method	#params	ACC (%)	SEN (%)	SPE (%)
AD vs. NC	Hosseini-Asl *et al*. 2018	~9,100 K	80.23 ± 4.57	82.97	77.60
	Korolev *et al*. 2017	~5,610 K	76.53 ± 7.47	79.38	73.93
	Rieke *et al*. 2018	~5,324 K	74.30 ± 6.34	76.21	72.50
	CNNs (Scratch)	~1,446 K	75.75 ± 6.08	81.45	73.16
	CAE	~1,446 K	85.24 ± 3.97	88.28	82.39
	ICAE	~371 K	86.60 ± 3.66	88.55	84.54
pMCI vs. NC	Hosseini-Asl *et al*. 2018	~9,100 K	71.01 ± 5.47	74.11	67.91
	Korolev *et al*. 2017	~5,610 K	68.85 ± 6.73	72.59	65.51
	Rieke *et al*. 2018	~5,324 K	69.95 ± 4.94	71.97	77.45
	CNNs (Scratch)	~1,446 K	68.12 ± 5.88	75.42	60.91
	CAE	~1,446 K	76.57 ± 3.96	82.66	70.03
	ICAE	~371 K	77.37 ± 3.55	81.03	74.07
sMCI vs. NC	Hosseini-Asl *et al*. 2018	~9,100 K	61.79 ± 4.44	61.98	61.61
	Korolev *et al*. 2017	~5,610 K	60.84 ± 5.23	62.23	59.56
	Rieke *et al*. 2018	~5,324 K	61.19 ± 4.89	63.04	69.37
	CNNs (Scratch)	~1,446 K	59.50 ± 5.84	61.47	57.74
	CAE	~1,446 K	62.83 ± 5.17	66.07	60.02
	ICAE	~371 K	63.04 ± 4.16	59.02	67.11
pMCI vs. AD	Hosseini-Asl *et al*. 2018	~9,100 K	60.83 ± 5.41	60.99	60.65
	Korolev *et al*. 2017	~5,610 K	59.24 ± 4.89	62.88	56.18
	Rieke *et al*. 2018	~5,324 K	60.53 ± 5.11	64.31	56.97
	CNNs (Scratch)	~1,446 K	55.62 ± 5.84	55.15	56.13
	CAE	~1,446 K	59.47 ± 6.32	60.00	58.92
	ICAE	~371 K	60.97 ± 5.33	64.53	56.13
sMCI vs. AD	Hosseini-Asl *et al*. 2018	~9,100 K	67.69 ± 6.41	69.58	65.63
	Korolev *et al*. 2017	~5,610 K	70.64 ± 5.83	70.08	71.28
	Rieke *et al*. 2018	~5,324 K	65.68 ± 4.19	61.33	69.44
	CNNs (Scratch)	~1,446 K	63.39 ± 7.14	66.31	60.12
	CAE	~1,446 K	74.68 ± 6.04	75.20	74.07
	ICAE	~371 K	75.06 ± 3.86	76.55	73.39

**Figure 9:**
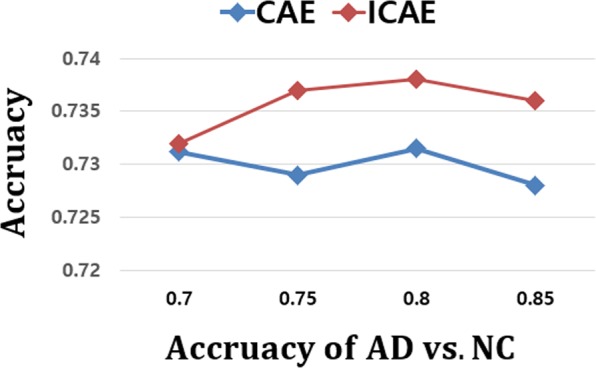
Comparison of transfer learning performances according to the accuracies of the source task. Target task: pMCI vs. sMCI; source task: AD vs. NC.

From a clinical perspective, the early autodiagnosis of MCI patients, who are at risk of progressing to having AD, is more crucial than the AD vs. NC problem for the effective treatment of AD. However, the classification of pMCI and sMCI is more challenging than that of AD and NC, since the anatomical changes that must be identified are subtler. In Table 3, the results of the CAE- and ICAE-based models showed poor accuracies of 60.69 and 61.39%, respectively, and we hypothesized that the complex patterns inherent in MCI subjects made it difficult to find proper initial weights for the network. To verify this assumption, we employed the pretrained AD vs. NC model to classify MCI subjects into pMCI and sMCI. To this end, pMCI and sMCI were considered as AD and NC, respectively, and an accuracy of 64.41% was observed, which was more favorable than that of the unsupervised learning method. Additionally, previous studies^30,31^ indicated that a source knowledge learned with an AD vs. NC task was effective for helping with pMCI detection. Motivated by this, we applied the supervised transfer learning technique to solve the pMCI vs. sMCI autodiagnosis problem. When implementing transfer learning, the convolutional layers of the 3D-CNNs were initialized using the pretrained weights of the AD/NC diagnostic model, and then, a joint supervised fine-tuning was performed to construct the network model for the pMCI/sMCI problem. In Table 3, the results clearly show that the transfer learning-based approach leads to substantially better accuracies, 73.23% and 73.95%, respectively, than those of the other approaches. In Fig. 9, we conducted an additional experiment to measure the correlation between the generalization capability of the source task and the transfer learning of the target task; as a result, no significant finding was observed, which shows that the high performance of the source task (AD vs. NC) does not guarantee better results for the target task (pMCI vs. sMCI).

**Table 3:** Classification performances of pMCI vs. sMCI (%).

Method	#parms	ACC (%)	SEN (%)	SPE (%)
Hosseini-Asl *et al*. 2018	~9,100 K	62.64 ± 5.53	65.15	60.53
Korolev *et al*. 2017	~5,610 K	61.22 ± 5.63	65.53	59.06
Rieke *et al*. 2018	~5,324 K	62.81 ± 6.15	66.93	58.85
CNNs (Scratch)	~1,446 K	60.64 ± 5.56	61.05	59.15
CAE	~1,446 K	60.69 ± 3.72	61.43	60.04
ICAE	~371 K	61.39 ± 4.76	62.56	60.41
CAE (Transfer learning)	~1,446 K	73.23 ± 4.21	74.96	71.53
ICAE (Transfer learning)	~371 K	73.95 ± 4.82	77.46	70.71

### Visualization results

**Figure 10:**
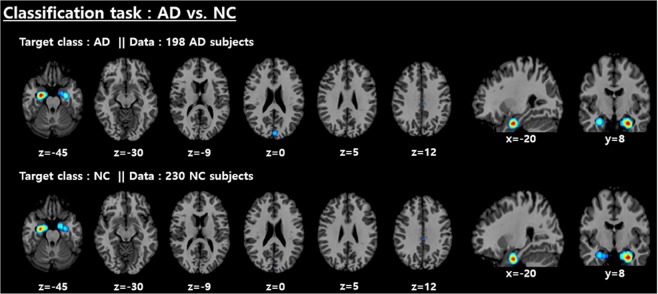
Distribution of discriminative regions in the classification of AD vs. NC.

**Figure 11:**
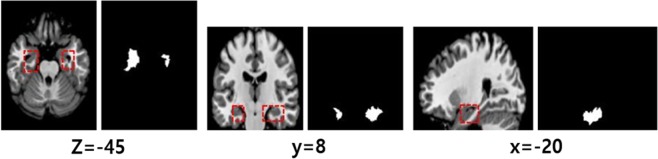
The most important brain areas that result in the CNNs classifying NC subjects into the AD category when implementing brain shrinkage simulation. Each left-hand image represents ROIs corresponding to the right binary supervoxel segment in the right-hand images (threshold 80% of max intensity).

We employed the CSV method^46^ to visualize features that make significant contributions to the determination of the given class. To this end, the validation data with the specific class were fed to the CSV method, and then the entirety of the saliency maps were linearly integrated and normalized to obtain the explainable saliency map using Algorithm 1. Subsequently, a highlighted salient region has statistical significance; this presentation may help readers intuitively understand the CNN model’s decision. The saliency maps for AD and NC subjects with their corresponding classes are shown in Fig. 10. The most discriminative features for the AD vs. NC classification task were mainly distributed around the medial temporal lobe. In detail, the left hippocampus is relatively more highlighted than other regions. Figure 11 shows the significant brain areas that result in CNNs classifying NC subjects into the AD category when implementing a brain shrinkage simulation. To this end, we simulated a brain shrinkage condition using a supervoxel-segmentation–wise occlusion method^22^ in which the original segment is replaced with a value of 0. After the simulation was performed on NC subjects, the most important brain areas calculated by accumulating the segments that resulted in the CNN’s original decision being overturned appear around the temporal lobe and include the amygdala and hippocampus.

**Figure 12:**
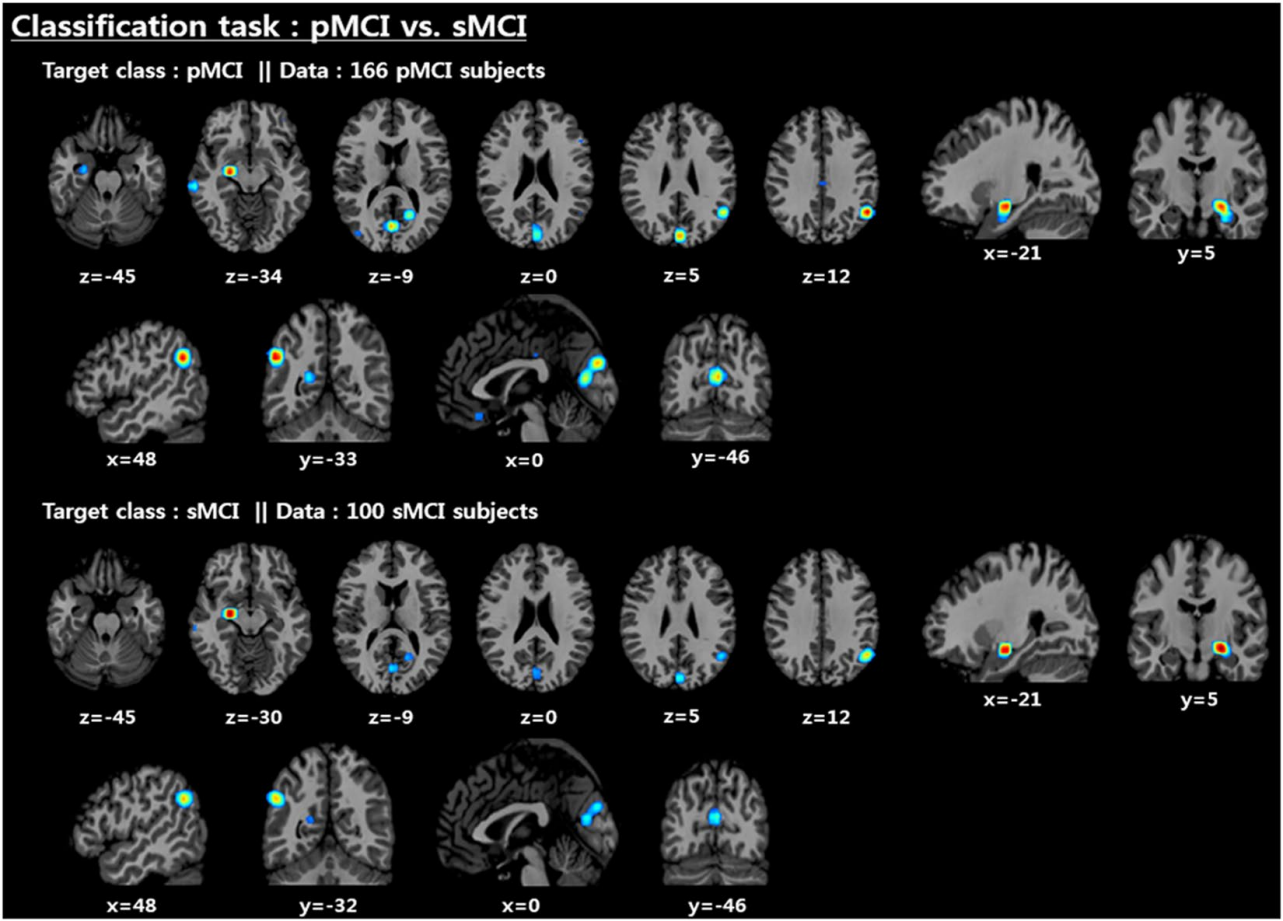
Distribution of discriminative regions in the classification of pMCI vs. sMCI.

As shown in Fig. 12, the left amygdala, angular gyrus, and precuneus were selected as the key biomarkers for the pMCI vs. sMCI classification problem. Unlike the AD vs. NC problem, the parts of the parietal lobe containing the angular gyrus and precuneus were additionally activated, and the left hippocampal region was deactivated, while its activation point was moved exactly to the left amygdala.

## Discussion

**Figure 13:**
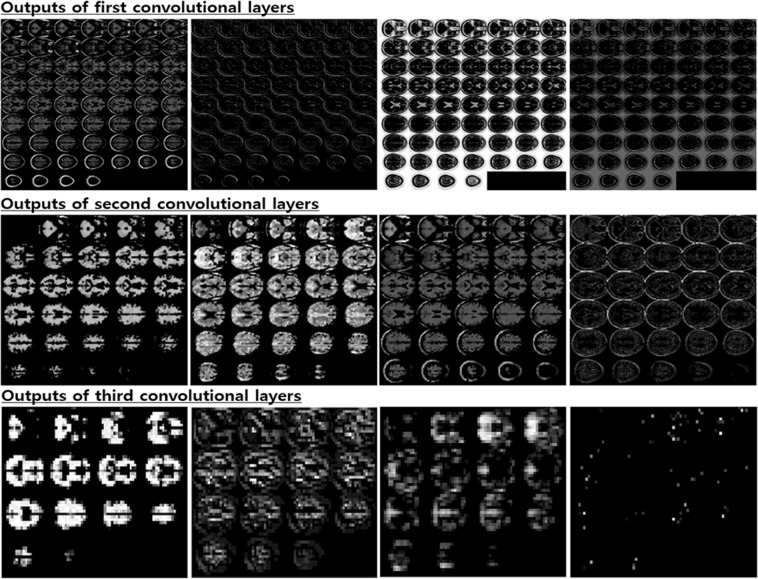
Visualization results of selected convolutional layer feature maps. From top to bottom: first, second and third convolutional layers.

The aim of this research was to study a way to encourage the end-to-end learning of a CNN-based model for AD/NC/MCI classification to ultimately have the capacity to obtain and analyze an explainable visualization map without human intervention. We devised a notion of the end-to-end learning hierarchy, and our work was built upon level 3 for which intensity and spatial normalization are only considered. We believe that this method is capable of maximizing the full ability of CNNs. A main advantage of CNNs compared to other machine learning methods is that the features can be automatically learned from raw data without any expert supervision. In this regard, output feature maps of each convolution layer automatically extracted by the CNNs are shown in Fig. 13. Some intense regions and segmentation clues can be observed in the first two convolution layers, and global feature maps that look like binary patterns can be extracted in the third convolution layer. The CNNs seem to conduct a level 2 preprocessing such as the white matter, CSF and skull segmentation automatically in the first two layers. Some sparse patterns are shown in the last layer, and we assume that they are related to the locations of important biomarkers. Additionally, the ability to achieve higher orders of abstraction and complexity relative to other machine learning methods renders CNNs better suited for detecting complex, scattered, and subtle patterns in data. Thus, this advantage may allow us to discover unknown biomarkers for neuroimaging analysis. Due to the endemic problem of neuroimaging caused by the high dimensionality, diversity, and lack of quantity associated with data, our methodology was strategically designed to overcome overfitting. To this end, we focused on encouraging network sparsity by using various regularization techniques to extract the sparse representations from the MRI scans. In the experimental results, it is worth noting that our strategy, in which network sparsity is reinforced by using regularization techniques (*L*_1_, *L*_2_, CAE-based, unsupervised learning and dropout with a high dropping ratio) dramatically boosts the performance of the network. Our strategy outperforms the existing methods^22,24,25^ and can be explained as follows. First, since no pretraining was considered in the baseline studies^22,24^, they suffer from biases caused by the random initialization of trainable weights, leading to performance decreases during the iterative performance evaluation. Similarly, this phenomenon was observed in our experiment for which scratch training was considered. Second, due to a lack of consideration of regularization techniques, the baseline model presented by Hosseini-Asl *et al*.^25^, which has an outdated architecture, seems to fail to achieve any favorable performance. We also observed that ICAE, even using fewer parameters, consistently improved the accuracy by 1~2% relative to CAE, and the strategy in which the network’s nonlinearity is encouraged appears to have a positive effect. Of particular interest, in the pMCI vs. sMCI classification task, we saw that the CAE-based unsupervised learning was not effective, and this may be attributed to the following two factors: first, the patterns inherent in MCI subjects are subtler; and second, the number of subjects is relatively smaller than that that of the AD vs. NC problem. It can sometimes be more effective to train the model on a simpler task and then move on to confront the more difficult task^34^. Inspired by this, we used the supervised transfer learning technique based on the pretrained AD vs. NC model, leading to performance increases of approximately 8~11%. In our transfer learning, this enabled the model to learn a representation of an MCI subject based on the discriminative biomarkers inherent in the problem of AD vs. NC, and this characteristic may be a reason for the favorable result. We are particularly sure that the knowledge transferring strategy can be applied to other neuroimaging domains that suffer from the same problem as we did initially when implementing end-to-end learning. After implementing the ADNI preprocessing protocol, only TPM-based spatial normalization was applied to encourage Level 3 end-to-end learning and produce an explainable saliency map. Although neuroimaging studies suffer from the abovementioned endemic data problems, the standardized spatial clue of the sMRI is a valuable characteristic that can be rarely found in most natural image processing methods having wide diversity, and we inferred that this characteristic was quite useful for CNN learning in the limited environment. To verify why the CNNs arrived at the classification decision given a particular instance and a target class, we produced a saliency map using the CSV approach. In the saliency maps shown in Figs. 10 and 12, the temporal and parietal lobes were selected by CNNs for accurate classification of AD, NC, and MCI subjects. These brain regions have previously been known to be closely related to dementia in many existing studies^26,27,43,44,50–56^. In particular, several previous studies^43,44^, references therein^26^ have found that the activity of the hippocampus is effective in the autodiagnosis of AD vs. NC. Consistent with previous studies, the hippocampus was a discriminative region in the classification of AD vs. NC in our study as well. Because of the dependency on and the significance of the hippocampal region, the CNNs appear to decide that it does not require other biomarkers to solve the AD vs. NC classification problem. Hippocampal volume is known to be a biomarker of Alzheimer’s disease that precedes cognitive impairment. For discriminating pMCI and sMCI, the CNNs seem to learn more complex representations than the AD vs. NC problem. Although the model achieved an accuracy of 73.95%, which is not high enough to be used in a real world situation, the saliency map included meaningful biomarkers, such as the amygdala, angular gyrus, and the precuneus, which have already been extensively reported in previous studies^51–55^. With regard to the temporal lobe, the hippocampus has received far less attention compared to the AD vs. NC problem, but the left amygdala was significantly activated. In this regard, the amygdala is affected early in AD, with neuropsychiatric symptoms leading to functional deficits; due to the early damage to the amygdala, neuropsychiatric symptoms are very common in mild stages of AD^57^. In addition, a relevant study^58^ that employs volumes of predefined ROI has reported that the amygdala is a more important biomarker than the hippocampus for detecting pMCI. Although the hippocampus has been a general feature for AD diagnosis, it may be inferred from this result that the hippocampus has no significant influence on minimizing the learning error of the CNNs for the classification of MCI subjects. Furthermore, our previous experiment, which considered the pretrained classifier gazing at the hippocampus region, yielded a relatively low accuracy of 64.41% for pMCI detection. However, after supervised transfer learning, the CNNs adjusted their gaze to several different biomarkers, leading to a performance increase of 8~11%. In terms of subject group, no significant findings were observed according to target class and subject group changes in both classification tasks. This demonstrates that the CNNs’ fixation is toward the same biomarkers, regardless of the type of subject group.

## Conclusions

In this section, we draw a number of conclusions. End-to-end learning without the use of hand-crafted features is possible, even in the endemic problems of neuroimaging where the training data are scarce and sample dimensionality is very high. Through this, we also produced an explainable visualization map intrinsically blocking the intervention of expert knowledge; this protocol is expected to serve as a differentiating method to find biomarkers related to AD or other neuropsychiatric disorders. The experimental results on the ADNI data demonstrated that our model achieved favorable performance and efficiency relative to current state-of-the-art models. However, this study has several limitations: First, as the number of subjects used for the training and test phases was still small for encouraging end-to-end learning, any performance improvement compared with the prior conventional models is limited. However, we believe that this approach shows a better generalization capability of learning models than hand-crafted–based approaches when a larger quantity of data is available in the future. Second, in the visualizing process performed by CSV, we had to approximate a coarse-scale saliency map on the template brain image because the exact localization of salient regions was limited. This limitation should be overcome in subsequent research. Despite these drawbacks, to the best of our knowledge, this is the first study to visualize most discriminant biomarkers related to AD/MCI without hand-crafted features by using the end-to-end learning of volumetric CNN architecture. Third, as there was gender difference between the several pairs of groups, this may have affected the outcome. In future work, extensive experiments involving network shape, hyperparameters, and different neuroimaging data may be required to determine an optimal network model.
