# Author Correction: Classification and Visualization of Alzheimer’s Disease using Volumetric Convolutional Neural Network and Transfer Learning

**DOI:** 10.1038/s41598-020-62490-1

**Published:** 2020-03-24

**Authors:** Kanghan Oh, Young-Chul Chung, Ko Woon Kim, Woo-Sung Kim, Il-Seok Oh

**Affiliations:** 1 Jeonbuk National University, Department of Computer Science and Engineering, Jeonju, 54896 Korea; 2 Research Institute of Clinical Medicine of Jeonbuk National University-Biomedical Research Institute of Jeonbuk National University Hospital, Jeonju, 54907 Korea; 3 Jeonbuk National University Medical School, Department of Psychiatry, Jeonju, 54907 Korea; 4 Jeonbuk National University Medical School, Department of Neurology, Jeonju, 54907 Korea

Correction to: *Scientific Reports* 10.1038/s41598-019-54548-6, published online 03 December 2019

This Article contains errors in references 5, 26, 36, 37 and 40, which were incorrectly given as:

Hayit, G., Bram van, G. & Ronald, M. S. Guest Editorial Deep Learning in Medical Imaging: Overview and Future Promise of an Exciting New Technique. IEEE Transactions on Medical Imaging. 35, 1153–1159 (2016).

Kilian, H. et al. Multimodal Hippocampal Subfield Grading For Alzheimer’s Disease Classification, https://doi.org/10.1101/293126 (2018).

Diederik, P. K. & Jimmy, B. Adam: A Method for Stochastic Optimization. In: ICLR. 2015 (2015).

Christian, S., Vincent, V., Sergey, L. & Zbigniew, W. Rethinking the Inception Architecture for Computer Vision. In: CVPR, https://doi.org/10.1109/CVPR.2016.308 (2016).

Loffe, S. & Szegedy, C. Batch normalization: accelerating deep network training by reducing internal covariate shift. In ICML. 448–456 (2015).

The correct references 5, 26, 36, 37 and 40 appear below as references 1–5, respectively.
